# Distribution of Artificial Radionuclides in Abandoned Cattle in the Evacuation Zone of the Fukushima Daiichi Nuclear Power Plant

**DOI:** 10.1371/journal.pone.0054312

**Published:** 2013-01-23

**Authors:** Tomokazu Fukuda, Yasushi Kino, Yasuyuki Abe, Hideaki Yamashiro, Yoshikazu Kuwahara, Hidekazu Nihei, Yosuke Sano, Ayumi Irisawa, Tsutomu Shimura, Motoi Fukumoto, Hisashi Shinoda, Yuichi Obata, Shin Saigusa, Tsutomu Sekine, Emiko Isogai, Manabu Fukumoto

**Affiliations:** 1 Graduate School of Agricultural Sciences, Tohoku University, Sendai, Miyagi, Japan; 2 Graduate School of Science, Tohoku University, Sendai, Miyagi, Japan; 3 Graduate School of Science and Engineering, Yamagata University, Tsuruoka, Yamagata, Japan; 4 Faculty of Agriculture, Niigata University, Niigata, Niigata, Japan; 5 Department of Pathology, Institute of Development, Aging and Cancer, Tohoku University, Sendai, Miyagi, Japan; 6 Graduate School of Dentistry, Tohoku University, Sendai, Miyagi, Japan; 7 RIKEN BioResource Center (BRC), Tsukuba, Ibaraki, Japan; 8 National Institute of Radiological Sciences, Chiba, Chiba, Japan; 9 Center for the Advancement of Higher Education, Tohoku University, Sendai, Miyagi, Japan; Kagoshima University Graduate School of Medical and Dental Sciences, Japan

## Abstract

The Fukushima Daiichi Nuclear Power Plant (FNPP) accident released large amounts of radioactive substances into the environment. In order to provide basic information for biokinetics of radionuclides and for dose assessment of internal exposure brought by the FNPP accident, we determined the activity concentration of radionuclides in the organs of 79 cattle within a 20-km radius around the FNPP. In all the specimens examined, deposition of Cesium-134 (^134^Cs, half-life: 2.065 y) and ^137^Cs (30.07 y) was observed. Furthermore, organ-specific deposition of radionuclides with relatively short half-lives was detected, such as silver-110m (^110m^Ag, 249.8 d) in the liver and tellurium-129m (^129m^Te, 33.6 d) in the kidney. Regression analysis showed a linear correlation between the radiocesium activity concentration in whole peripheral blood (PB) and that in each organ. The resulting slopes were organ dependent with the maximum value of 21.3 being obtained for skeletal muscles (R^2^ = 0.83, standard error (SE) = 0.76). Thus, the activity concentration of ^134^ Cs and ^137^Cs in an organ can be estimated from that in PB. The level of radioactive cesium in the organs of fetus and infants were 1.19-fold (R^2^ = 0.62, SE = 0.12), and 1.51-fold (R^2^ = 0.70, SE = 0.09) higher than that of the corresponding maternal organ, respectively. Furthermore, radiocesium activity concentration in organs was found to be dependent on the feeding conditions and the geographic location of the cattle. This study is the first to reveal the detailed systemic distribution of radionuclides in cattle attributed to the FNPP accident.

## Introduction

The accident at the Fukushima Daiichi Nuclear Power Plant (FNPP) discharged volatile artificial radionuclides such as ^129m^Te, ^132^Te, ^131^I, ^132^I, ^133^Xe, ^134^Cs, ^136^Cs and ^137^Cs into the environment [1]–[5]. The details of the timing and causes of the radioactive releases have been monitored by Tokyo Electric Power Company (TEPCO) (http://www.tepco.co.jp/nu/fukushima-np/f1/index-e.html). The Ministry of Education, Culture, Sports, Science and Technology (MEXT), Japan, and the U.S. Department of Energy (U.S. DOE) examined the airborne dose rate 1 m above the ground surface within 80 km from the FNPP (http://radioactivity.mext.go.jp/en/). In order to assess biological effects of radiation exposure, evaluations of both external and internal exposure are important. While a variety of data pertaining to external exposure are available, only limited data of the internal distribution of radionuclides and their activity are available. Deposits and internal exposure of radionuclides are dependent on the metabolism and biokinetics of radionuclides, which are not fully understood in animals. As of April 22, 2011, the evacuation zone was set to within a 20-km radius surrounding the FNPP, and approximately 3,400 cows, 31,500 pigs, and 630,000 chickens were left behind in this area. On May 12, 2011, the Japanese government ordered Fukushima Prefecture to euthanize the cattle in the evacuation zone. Recently, Calabrese indicated the importance of risk assessment for chronic radiocesium exposure based on the situation at Fukushima in Japan and emphasized the limitation for the assessment of risk from radiocesium intake due to the absence of animal model chronic bioassays [6]. Almost all of the cattle abandoned in the evacuation zone can be recognized individually by their ear-tag and their individual histories are easily obtainable. We therefore thought that those would be ideal as an animal model for assessing chronic exposure to radionuclides after the FNPP accident. In order to provide basic information for biokinetics of radionuclides and for dose assessment of internal exposure, we evaluated gamma-ray emitting artificial radionuclides in multiple organs of the cattle and analyzed their organ specificity and metabolism.

## Results and Discussion

Between August 29 and November 15, 2011, we collected 79 cattle in total, 27 of which were from Minami-soma city located north and 52 from Kawauchi village located southwest of the FNPP. Both places are within the evacuation zone between 10- and 20-km radius of the FNPP. The cattle included 63 adult females (3 of which were pregnant), 10 male calves, and 3 female calves. The age of the cattle without the ear-tag was estimated by its height and body size. We classified the cattle as infant if it was younger than 6 months old.

Typical gamma-ray spectra obtained from muscle, liver, and kidney are shown in Figure S1. In these spectra, either of photopeaks from ^134^Cs, ^137^Cs, ^110m^Ag, and ^129m^Te was observed. In the control animals, which were housed in Hokkaido Prefecture (northern edge of Japan, 630 km from FNPP), we could not detect any photopeaks of ^134^Cs, ^137^Cs, ^110m^Ag, and ^129m^Te (data not shown). We also determined the limit of detection based on the background measurement (see “Materials and Methods”). From these data, we concluded that the detected radionuclides, such as ^134^Cs, ^137^Cs, ^110m^Ag, and ^129m^Te, in the abandoned cattle were attributable to environmental contamination by the nuclear fallout from the FNPP accident.

Based on the count rate from each cattle organ, we calculated the concentration of these radionuclides, as listed in Table 1. All the measurements were decay-corrected to the day of major release, March 15, 2011 (see the section on decay correction in Materials and Methods). All specimens including peripheral blood (PB) obtained from abandoned cattle showed deposition of two radionuclides, ^134^Cs and ^137^Cs. The measured radioactivity of ^134^Cs was similar to that of ^137^Cs after decay correction in all samples examined. Therefore, both isotopes of radiocesium are hereafter represented by ^137^Cs. Muscle tissues showed the highest deposition of ^137^Cs and no statistical difference of ^137^Cs activity concentration was observed among three representative positions of the cattle muscle (*Longissimus*, *Biceps femoris*, and *Masseter* muscle). We thus classified these three muscles into one category, “skeletal muscle,” in the subsequent regression analysis between PB and organs.

**Table 1:** Activity concentration of ^134^Cs, ^137^Cs, ^110m^Ag and ^129m^Te in cattle organs and peripheral blood.

	Cs-134 (Bq/kg)^a^			Cs-137 (Bq/kg)^a^			Ag-110m (Bq/kg)^a^			Te-129m (Bq/kg)^a^		
	mean ± SD^b^		num^c^	mean ± SD^b^		num^c^	mean ± SD^b^		num^c^	mean ± SD^b^		num^c^
Longissimus muscle	592	±398	48	611	±416	48		ND^d^			ND	
Biceps femoris muscle	637	±420	38	665	±442	38		ND			ND	
Masseter muscle	579	±359	19	606	±373	19		ND			ND	
Neck muscle	549	±300	6	568	±327	6		ND			ND	
Diaphragm	272	±206	12	289	±233	12		ND			ND	
Tongue	584	±310	17	619	±334	17		ND			ND	
Heart	301	±176	30	311	±187	30		ND			ND	
Liver	198	±145	47	207	±154	47	177	±176	47/47^e^		ND	
Kidney	344	±249	29	361	±264	29		ND		7,000	±6,000	18/29^e^
Lung	272	±186	35	275	±183	35		ND			ND	
Spleen	182	±98	20	190	±106	20		ND			ND	
Thyroid gland	200	±231	8	192	±207	8		ND			ND	
Submandibular gland	162	±101	11	178	±114	11		ND			ND	
Mammary gland	54	±60	3	54	±55	3		ND			ND	
Uterus	128	±114	6	143	±125	6		ND			ND	
Urinary bladder	186	±80	5	210	±94	5		ND			ND	
Brain	119	±36	3	123	±38	3		ND			ND	
Eye	103	±55	11	110	±62	11		ND			ND	
Blood	24	±20	51	25	±19	51	5.2	±3.7	5/51^e^		ND	

a Decay correction was made to the day major release of radionuclides, March 15, 2011.

b SD: the standard deviation.

c num: the number of the samples positive for the deposition of radionucleides.

d ND: not detectable.

e The Number of positive samples/the number of tested samples. All the samples were positive for ^134^Cs and ^137^Cs.

In the liver (100%: 47/47 animals) and PB (9.8%: 5/51 animals), ^110m^Ag (half-life: 249.8 d) was detected (Table 1). Furthermore, kidney (62%: 18/29 animals) showed the deposition of ^129m^Te, despite its short half-life (33.6 d), whereas other specimens including PB did not.

**Figure 1:**
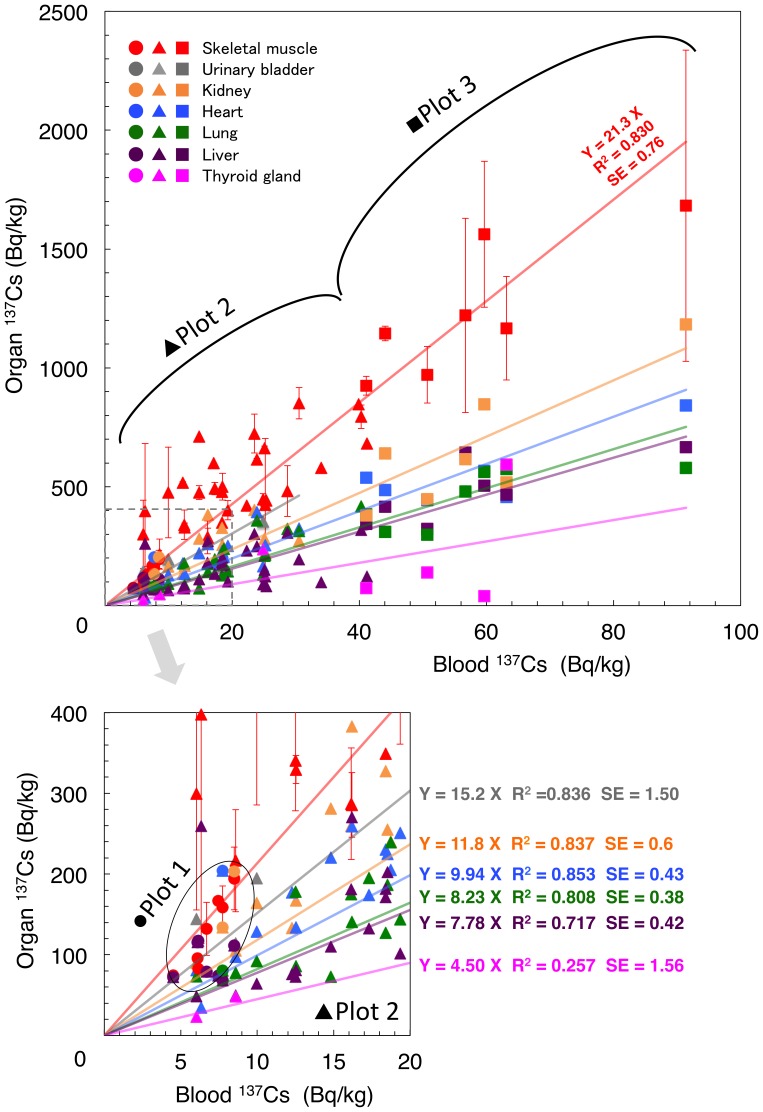
Correlation of ^137^Cs activity concentration between peripheral blood (PB) and organs. Cattle were captured in Plots 1(circle), 2 (triangle), and 3 (square). Cattle from the same plot were enclosed by black marking. Inset: Cattle whose ^137^Cs radiation concentration in PB was lower than 20 Bq/kg. All those from Plot 1 and part of Plot 2 were included.

Regression analysis revealed linear correlations of ^137^Cs activity concentration between PB and organs. The slope was dependent on each organ examined (Figure 1), indicating that radiocesium activity concentrations in the organs can be estimated from the blood levels. The activity concentration level of ^137^Cs in the skeletal muscle was the highest among all of the organs examined and was 21.3-fold greater (standard error (SE) = 0.76) than that of PB. The difference of the regression slopes between the skeletal muscle and other organs was significant (t-test, Table S1). Interestingly, ^137^Cs activity concentration in the heart was significantly lower than that in skeletal muscles despite the fact that the heart is composed of striated muscle cells.

Cattle were divided into 3 groups in accordance with the plot in which they were caught. Plots 1 and 3 were in Minami-soma city located to the north and plot 2 in Kawauchi village located to the southwest of the FNPP, respectively. Cattle in Plot 1 were kept in a stall barn after the FNPP accident, and they were fed with radionuclide-free pasture grass and supplied with radiation contaminated rainwater until sacrifice. Cattle in Plots 2 and 3 were unleashed and were freely allowed to graze on contaminated grass after the accident. The profile of rdiocesium activity concentration could be divided into 3 groups in accordance with the Plot where the cattle were caught (Figure 1 and Table S2). Although Plots 1 and 3 were the same city, the feeding conditions were different. The level of ^137^Cs activity concentration in soil samples between Plot 2 and Plot 3 was not so different (Table S3) and was also reportedly quite similar [7]. However, ^137^Cs activity concentration in PB of cattle in Plot 3 was the highest and that in Plot 1 was the lowest in this study (comparison of each group, p<0.01, t-test). These indicate that the activity concentration of internally deposited radionuclides is largely influenced by feeding conditions as well as geographic conditions of the cattle farm.

**Figure 2:**
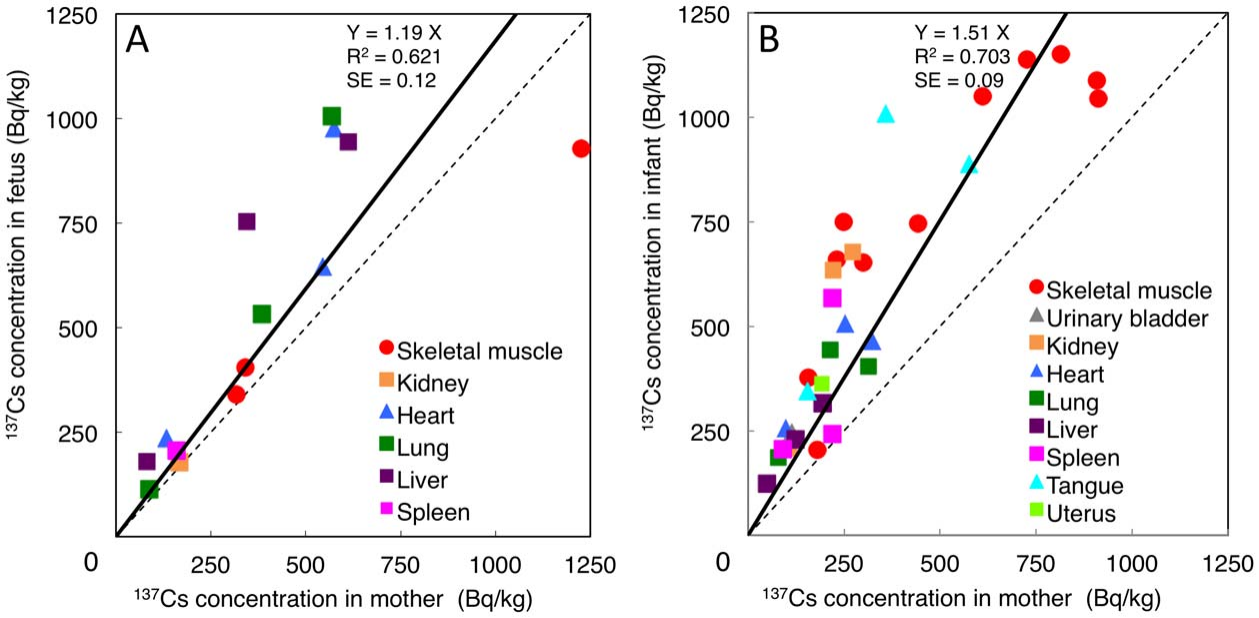
Comparison of ^137^Cs activity concentration between mother and offspring. **A.** Organ ^137^Cs activity concentration between 3 pairs of mother and her fetus. **B.** Organ ^137^Cs activity concentration between 3 pairs of mother and her child. The dashed line indicates the slope at which ^137^Cs activity concentration in organs is equal between mothers and their corresponding offsprings. Points above this line indicate that offspring ^137^Cs activity concentration in an organ was higher than that of the mother. The bold line is the regression line obtained from all organs.

The transfer of radionuclides from mother to fetus is one of the major concerns of exposure to internal radiation. As mentioned above, we had collected 3 pregnant cows. The comparison between radiocesium activity concentration in the fetus and the mother is shown in Figure 2A. Most symbols lay on the upper part of the dashed equality line (fetus side). The regression analysis of all tested organs showed that the activity concentration of cesium was 1.19 times higher (R^2^ = 0.62, SE = 0.12) in fetal organs than in the corresponding maternal organs. Cesium is considered to transfer freely between mother and fetus and is assumed to be uniformly distributed throughout all the tissues of the fetus [8]. These observations indicate that radiocesium is more concentrated in the fetus than in the mother. Although Silver and Tellurium are transplacental [9]–[11], neither ^110m^Ag nor ^129m^Te was detectable in the fetal organs examined, indicating that these radionuclides were efficiently captured by the mother's organs and were not delivered to the fetus. In order to understand fetal biokinetics of each radionuclide we tried to collect PB from the fetus. However, we could not obtain sufficient quantities to enable the measurement of radioactivity.

Before the cattle were euthanized, we noticed 3 mother and infant pairs in Plot 2. We confirmed that these infants were born after the FNPP accident and that they were being weaned at the time. As shown in Figure 2B, most of the points lie on the upper side of the dashed equality line (infant side). The regression analysis showed that radiocesium activity concentration was 1.51 times higher in the infant organs than in the corresponding maternal organs (R^2^ = 0.70, SE = 0.09). Therefore, we concluded that the deposition of ^137^Cs in infant organs is correlated with that in the corresponding maternal organs but is higher than that in maternal ones. Shorter half-lives of retention are adopted for infants and children than for adults [12]. Inaba *et al.* mentioned that water and electrolyte metabolism should differ considerably between newborn and adult, and that potassium contents of the feeding might affect the radiocesium activity concentration [13]. We do not have any data regarding the proportion attributed to milk and grass which the infants were taking at the time of the sacrifice.

In our data, the thyroid showed lower ^137^Cs deposition compared with other visceral organs (Figures 1 and 2B). Bandazhevsky previously reported that the highest accumulation of radiocesium was found in the endocrine glands, in particular, the thyroid, in humans [14]. Although we need to consider the species difference between humans and cattle, radiocesium is suggested to have little impact on thyroid carcinogenesis.

Chronic inflammation and the development of proliferative atypical cells of the bladder uroepithelium in people living in ^137^Cs-contaminated areas in Ukraine have previously been reported [15]. The urinary bladder showed relatively high ^137^Cs accumulation in this study (Figure 1). In our observation, so far, we could not find any abnormalities at the gloss appearance level in the bladder.

**Figure 3:**
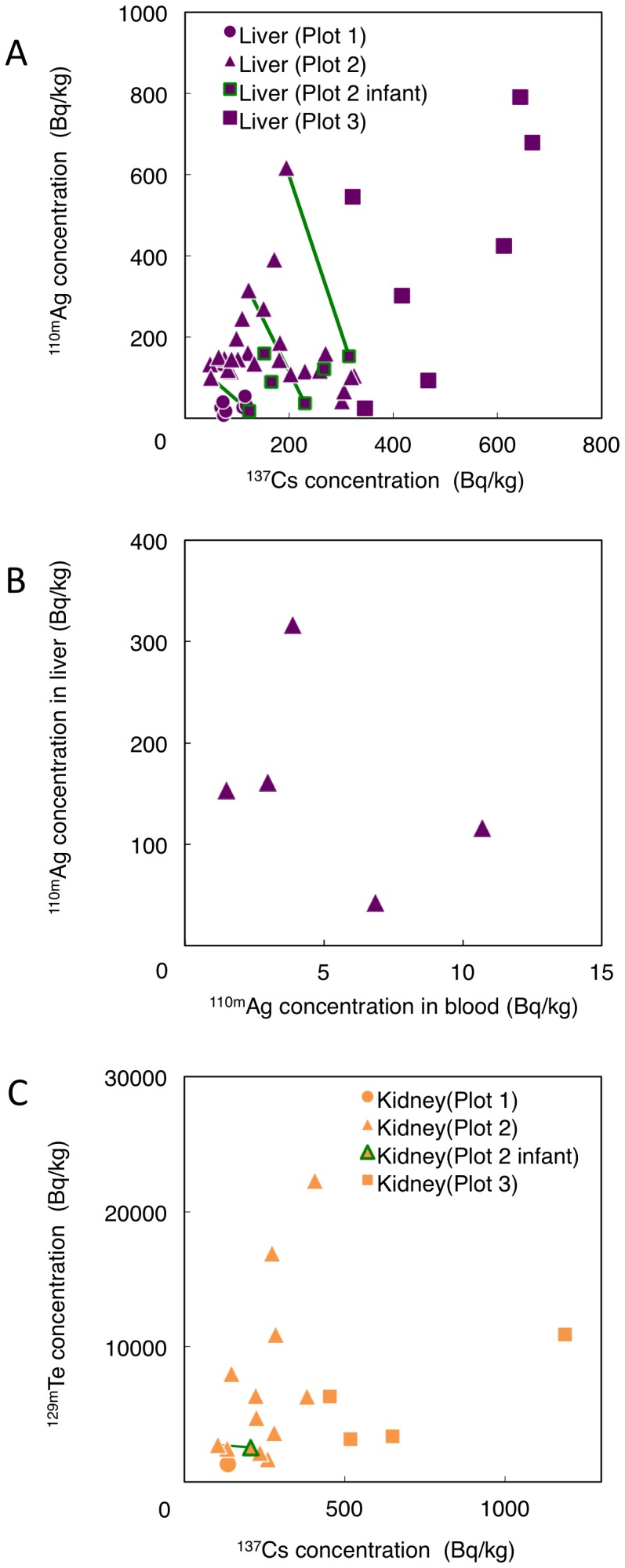
Activity concentration of ^110m^Ag and ^129m^Te.

Radioactive ^110m^Ag is not a fission product but is formed by the neutron capture of stable ^109^Ag. We detected ^110m^Ag in the liver of all of the cattle except for fetuses examined (Table 1 and Figure 3A). The ratio of deposited radioactivity concentration of ^110m^Ag to ^137^Cs in the soil of Plot 2 and Plot 3 was lower than 0.5% and that in the grass of Plot 3 was lower than 5% (Table S3). The value in the soil was consistent with the distribution map of radiation doses by MEXT (http://radioactivity.mext.go.jp/old/en/1750/2011/10/1750_1031e_2.pdf) (MEXT Dose Map) as of June 14, 2011. In the current study, ^110m^Ag activity concentration in the liver did not show Plot dependent difference or association with ^137^Cs activity concentration (Figure 3A). Both the human evidence and the animal studies indicate substantial deposition of silver in the liver but the retention rate is influenced by the route of intake [12]. It is reported that the liver deposition of ^110m^Ag in sheep and its transfer coefficient to the liver was higher than that of ^137^Cs in the Chernobyl nuclear accident [16]. These data indicate that the transfer coefficient of ^110m^Ag to the liver is higher than that of ^137^Cs. Furthermore, post-mortem data on the distribution of ^110m^Ag in a patient 195 days after injection showed the highest uptake in the liver (40%) among all organs [17]. There was no relationship between the activity concentration of ^110m^Ag in PB and in the liver (Figure 3B). Danscher *et al.* reported that silver predominantly accumulates in lysosome-associated tissues, such as lymph nodes, liver, kidneys and the central nervous system after silver administration in rats and mice. Furthermore, they showed the intense accumulation of silver in Kupper cells of the liver [18]. From these cumulative data and this study, we concluded that the liver is the primary target organ for ^110m^Ag deposition.

We clearly detected the radioactivity of ^129m^Te in the cattle kidney (Table 1), although the measurement was performed 7 months after the FNPP accident. We concluded that the kidney is the target organ of ^129m^Te deposition, based on its relatively short half-life (^129m^Te, 33.6 d). The activity concentration of ^129m^Te was mainly classified into two groups: Plots 2 and 3 (Figure 3C). The ratio of ^129m^Te/^137^Cs activity concentration in the kidney of some cattle from Plot 2 was higher than that from Plot 3. As of March 15, the ratio of soil activity concentration of ^129m^Te to ^137^Cs was 0.34 in Plot 2 and 1.41 in Plot 3, respectively [7]. This contradiction needs further investigation.

After the FNPP accident, a large amount of ^132^Te was released into the environment. At first a higher activity concentration of ^132^Te than ^129m^Te was detected in the soil of the evacuation zone [7]. The deposition of ^129m^Te in the kidney suggests that radioactive ^132^Te also accumulated in the kidney shortly after the FNPP accident. The half-life of ^132^Te is 3.2 days and its decay product is radioactive ^132^I, which is thyroid tropic. A previous study reported that radioactive tellurium that is orally administered to cows concentrates in the thyroid more than in most other tissues [19]. These results suggest that we need to pay more attention to ^132^Te as well as ^131^I in assessing health risk to the thyroid.

Currently, we are further collecting tissues from animals, including cattle, in the evacuation zone in order to construct a tissue bank which represents a variety of species. As the first stage, we are going to make dose assessments of deposited radionuclides in animals. Microscopic examinations of necropsied animals are also underway to find lesions that could be directly attributed to the effect of ionizing radiation. Our study is the first report on organ-specific deposits of various gamma-ray emitting radionuclides in cattle after the FNPP accident, and should contribute to improvement in public health and radiation safety.

## Materials and Methods

### Ethics

This study is one of the national projects associated with the Great East Japan Earthquake and has been entirely endorsed and supported by the Japanese government through the Ministry of Education, Culture, Sports, Science and Technology, Japan. The Japanese government ordered Fukushima Prefecture to euthanize cattle in the evacuation zone on May 12, 2011 to prevent radio-contaminated beef products entering the human food chain. We collected organs and tissues from the euthanized cattle by the combined unit of veterinary doctors belonging to the Livestock Hygiene Service Center (LHSC) of Fukushima Prefecture and those belonging to the Ministry of Agriculture, Forestry and Fisheries, Japan. Cattle were sacrificed by these veterinarians by the following method according to the Regulation for Animal Experiments and Related Activities at Tohoku University (Regulation No 122) briefly described as below. The owner of each cattle was identified by the ear tag of the cattle and informed consent from the owner was obtained by the veterinary doctors of Fukushima Prefecture. The procedure of euthanasia was entirely carried out by the veterinary doctors of LHSC. The cattle used as negative control for radioactive substances were used for practical training of anatomy for students at Rakuno Gakuen University, Hokkaido, Japan. Use of the cattle for this study was approved by the Ethics Committee for Animal Experiments of Rakuno Gakuen University. These cattle were sacrificed by exanguination from the jugular vein in their unconscious state by a pentobarbital overdose via intramuscular injection of hypnotics (Xylazine hydrochloride, 0.2 mg/kg), then PB was collected from the jugular vein, after which Pentobarbital (2 mg/kg) and Suxamethonium were injected.

### Collection of specimens

Between August 29 and November 15, 2011, we obtained PB and organ samples from cattle collected in the evacuation zone of the FNPP. All cattle born before the FNPP accident had the ear tag with an individually unique 10-digit number for identification, however, those born after the FNPP accident did not. After tranquilization by intramuscular injection of hypnotics (Xylazine hydrochloride, 0.2 mg/kg), PB was collected from the jugular vein and then Pentobarbital (2 mg/kg) and Suxamethonium chloride (1 mg/kg) were injected. We collected tissues within 1 hr after veterinary doctors euthanized the cattle. Because dissection was carried out in an open field with limited time being spent in the evacuation zone, the number of organs extirpated differed among individual cattle, for example, only blood samples and a single muscle sample could be obtained from some cattle. PB and organ samples were preserved at −20°C until radioactivity was measured. The number of organ samples analyzed is shown in Table 1. Soil and grass samples were collected at the place where the cattle were caught. Soil samples were taken in a square 30×30 cm from the surface to the depth of 1 cm. Radioactivity concentration was calculated into kBq/m^2^ by [measured radiation concentration (Bq/kg)]×[measured density (kg/m^3^)]×[depth (0.01 m)]. We carefully selected grasses with bite marks of cattle if possible and radioactivity concentration was calculated into Bq/wet weight.

### Collection of control specimens

In Hokkaido Prefecture (northern edge of Japan, 630 km from FNPP), we collected samples from 2 male and 1 female cows housed in this area that were around 17 days of age. Furthermore, their mothers were supplied with radionuclide-free feed. Organs dissected from these cattle were used as negative control, which are free for radionuclides from FNPP. After the cattle were euthanized, the same cattle organs as listed in the previous section were dissected. The radionuclides in the control organs were measured by the same method as that for the abandoned cattle, as described in the next section.

### Measurements of radioactivity

The radioactivity of the samples was determined by gamma-ray spectrometry using three HPGe detectors (Ortec Co., USA). The measurement time varied from 3,600 to 200,000 s depending on the radioactivity of the samples. The efficiency was determined by measuring mixed sources of ^152^Eu and ^137^Cs. An aliquot (200 ml) of the mixed source was diluted with appropriate amounts of water, and superabsorbent polymer was added to the mixture to make a gel standard source. The gel source was used as a mock sample to imitate tissue samples, and several types of gel sources were prepared to cover a weight range from 0.5 to 110 g. Aqueous solutions of ^152^Eu and ^137^Cs were used to determine the efficiency for liquid samples such as PB. Samples were homogenized and sealed in polyethylene vials (1–100 ml) depending on their mass. A nuclide was identified when its characteristic photopeaks of greater than 3σ above the baseline were observed in the spectrum. In addition, we determined the radioactivity detection limit from the background level: it was found to be 0.1–0.2 Bq/kg for organs from which we could obtain sufficient quantities of samples to fill a 100-ml vial and 0.7–0.8 Bq/kg for relatively tiny organs (less than 10 g in weight), such as the thyroid gland.

### Decay correction

Presenting radioactivity measured as *a* (Bq), the activity after the decay correction is given by *a_0_* (Bq). We can obtain the number of days, *t* from March 15, 2011 to the date of the measurement. The half-life, *T* is 250 days for ^110m^Ag, 34 days for ^129m^Te, 754 days for ^134^ Cs and 11,016 days for ^137^Cs.

The activity after the decay correction is given by:
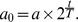



### Statistic analysis

Linear regression analysis was performed to determine the relationship of two variables and to evaluate the regression line and standard error. The difference between the regression slopes of two groups was found to be significant in case p<0.05 with the standard t-test.

## Supporting Information

Figure S1 **Representative detected photopeaks for internal radionuclides in organs of a cattle.** **A.** Both peaks form ^134^Cs and ^137^Cs are highest in the muscle among organs meaured. **B.** Characteristic peaks to ^110m^Ag are observed in the liver but not in the muscle or the kidney. **C.** A peak from ^129m^Te is obsereved in the kidney but not in the muscle or the liver.
(TIF)

Table S1 **P values for null hypothesis test between regression slopes of two different organs determined (t-test).** * The value of slopes: Skeletal muscle>Urinary bladder>Kidney>Heart>Lung>Liver>Thyroid gland
(TIFF)

Table S2 **Concentration of 134Cs and 137Cs in cattle organs.** ^a^ Skeltal muscles: Longissimus muscle, Biceps femoris muscle and Masseter muscle combined. ^b^SD: Standard deviation. ^c^ num: the number of the samples positive for the deposition of radionucleides.
(TIFF)

Table S3 **Radioactivity concentration in the soil (kBq/m2) and in the grass (Bq/kg).** ^a^JPG: Japanese pampas grass. ^a^ND: not detectable.
(TIFF)
