# Supplementary figures and images for: Distribution of Artificial Radionuclides in Abandoned Cattle in the Evacuation Zone of the Fukushima Daiichi Nuclear Power Plant
Source: PLoS One. 2013 Jan 23;8(1):e54312. doi: 10.1371/journal.pone.0054312 (PMC3553152; doi:10.1371/journal.pone.0054312)

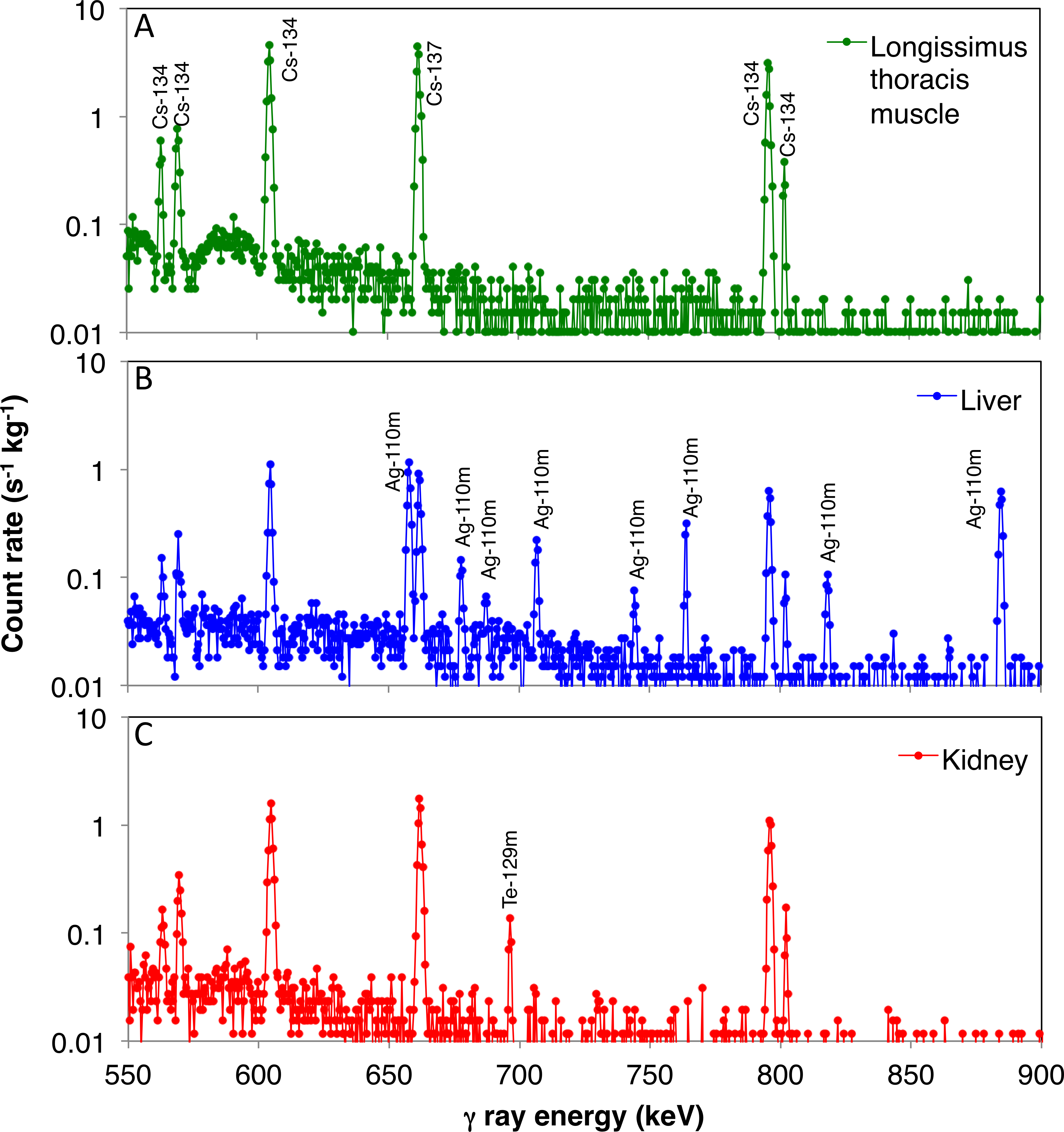

Supplement: Figure S1 — Representative detected photopeaks for internal radionuclides in organs of a cattle. A. Both peaks form 134Cs and 137Cs are highest in the muscle among organs meaured. B. Characteristic peaks to 110mAg are observed in the liver but not in the muscle or the kidney. C. A peak from 129mTe is obsereved in the kidney but not in the muscle or the liver. (TIF) [file pone.0054312.s001.tif]

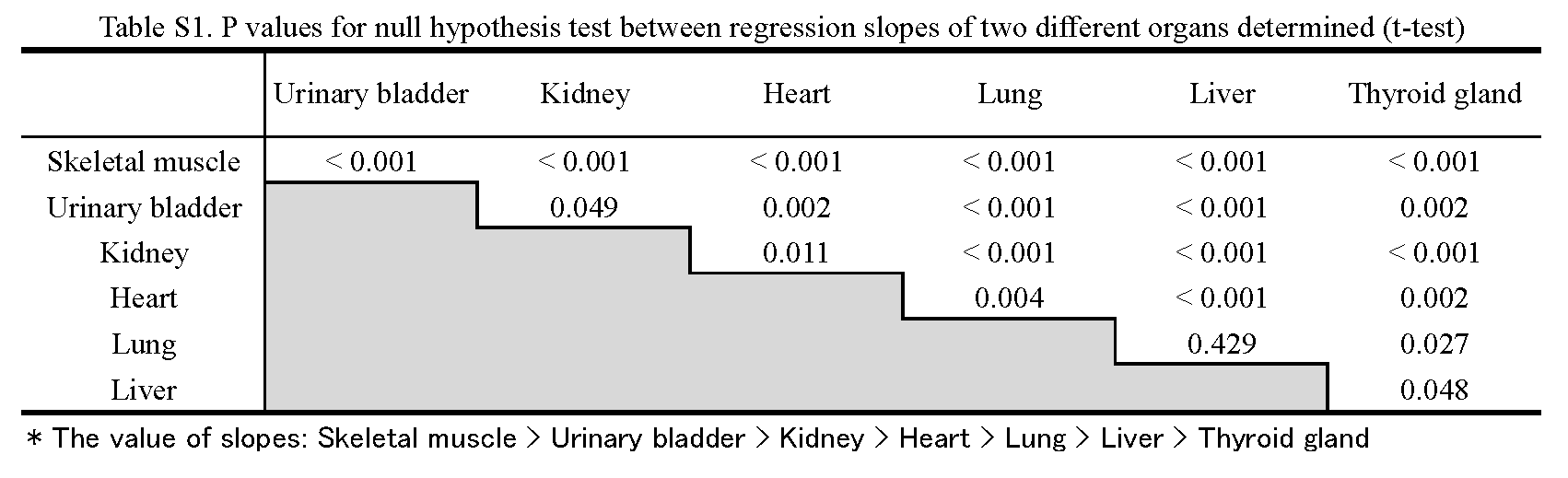

Supplement: Table S1 — P values for null hypothesis test between regression slopes of two different organs determined (t-test). * The value of slopes: Skeletal muscle>Urinary bladder>Kidney>Heart>Lung>Liver>Thyroid gland (TIFF) [file pone.0054312.s002.tiff]
